# The impact of lactic acid bacteria inoculation on the fermentation and metabolomic dynamics of indigenous Beijing douzhi microbial communities

**DOI:** 10.3389/fmicb.2024.1435834

**Published:** 2024-07-30

**Authors:** Dong Han, Xinyu Bao, Yanfang Wang, Xiaohong Liao, Ke Wang, Jian Chen, Xiaolong Li, Zhennai Yang, Yanbo Wang

**Affiliations:** ^1^Beijing Engineering and Technology Research Center of Food Additives, School of Food and Health, Beijing Technology and Business University, Beijing, China; ^2^Key Laboratory of Food Bioengineering (China National Light Industry), College of Food Science and Nutritional Engineering, China Agricultural University, Beijing, China; ^3^China National Light Industry Council, Beijing, China; ^4^Food Safety Key Laboratory of Zhejiang Province, School of Food Science and Biotechnology, Zhejiang Gongshang University, Hangzhou, China

**Keywords:** fermented foods, lactic acid bacteria, microbial community, Beijing douzhi, metabolomics

## Abstract

**Background:**

Douzhi, a traditional Chinese fermented beverage, features microbial communities primarily composed of lactic acid bacteria (LAB). As fermented foods continue to gain recognition and popularity, douzhi is attracting growing interest. However, investigation of the critical aspects of douzhi’s fermentation processes, including fermentation characteristics and microbial community dynamics, remains vital for enhancing food safety and quality for douzhi, as well as for similar fermented food products.

**Method:**

In this study, we collected douzhi microbial communities from four chain stores, using them as fermentation starter cultures. The microbial dynamics of the fermentation were analyzed, focusing on the inoculation of LAB strains and the transition from a mung bean-based matrix to skimmed milk. The metabolomic profiles of the fermented mung bean matrices were also studied.

**Results:**

Douzhi samples obtained from representative chain stores were found to be overwhelmingly dominated by LAB. When inoculated along with the douzhi community, both LAB strains exhibited notable and substantial reductions in the pH value of the designated mung bean matrices compared to those inoculated indigenous microbiota. Specifically, *Lactiplantibacillus plantarum* CGMCC 1.1856 retained its population, whereas *Pediococcus pentosaceus* CGMCC 1.2695 exhibited a decrease in relative abundance. Using skimmed milk as a fermentation substrate instead of the mung bean matrix resulted in significant shifts in microbial communities, particularly leading to an increase in *Escherichia* sp. The metagenomic analyses and functional predictions illustrated that various metabolic functions were enhanced during the fermentation process due to LAB inoculation. The liquid chromatography–mass spectrometry based metabolomic analysis revealed that the inoculation of *Lactiplantibacillus plantarum* and *Pediococcus pentosaceus* in mung bean matrix did not introduce new metabolites but significantly altered the concentration and profile of existing metabolites, especially increased low molecular carbohydrates, which may enhance the nutritional potential of the fermented product.

**Discussion:**

This study examines the microbial dynamics of douzhi microbiota fermentation, emphasizing the role of lactic acid bacteria in enhancing fermentation activity and metabolite profiles. These insights contribute to improving manufacturing processes and ensuring the safety and quality of douzhi and similar fermented foods.

## 1 Introduction

Fermented foods, representing a deep-rooted culinary tradition of mankind that transcends time, geography, ethnicities, and culture, which can be traced back to the need of preservation of perishable ingredients (Ray and Joshi, 2014; Tamang et al., 2020). It exerts crucial role in preserving and enhancing designated foods ingredients until today. According to studies, traditional fermented foods are estimated to comprise up to one-third of the human dietary intake nowadays (Campbell-Platt, 1994; Marco et al., 2021; Mukherjee et al., 2022). Nevertheless, the quality and safety control of both spontaneously fermented foods and those inoculated with starters remains an enduring quest, especially given the ongoing evolution of the modern food industry. This encompasses not only the dynamics within food supply chain, such as the expansion of production scale which demands for extended transportation and storage, but also related technological advancements, including widespread production automation and the utilization of bioengineered strains (Tamang et al., 2020; Galimberti et al., 2021). The concept of “fermented food” was recently defined as “foods made through desired microbial growth and enzymatic conversions of food components” by the International Scientific Association for Probiotics and Prebiotics (ISAPP), highlighting the intricate fermentation processes involving microbial proliferation and the corresponding enzymatic activities (Marco et al., 2021). Nowadays, fermented food production comprises both large-scale, highly industrialized operations with advanced microbiological control technologies and indigenous small-scale, even household-level, fermentation processes. These indigenous small-scale processes are particularly notable in underdeveloped regions, where they play a significant role in local food production and preservation (Mukherjee et al., 2022; Ramos et al., 2023). Well-controlled fermentation processes, often incorporating established starter cultures and rigorous hygiene measures, confer significant advantages to consumers and markets. While in smaller-scale fermented food productions, the establishment of a secured microbial ecosystem within the production unit, dependent on the quality of raw materials and the expertise of the producer, plays a pivotal role in ensuring the production of safe and high-quality products, which help to mitigate the risk of compromising food safety and the occurrence of foodborne disease outbreaks (Mukherjee et al., 2022; Skowron et al., 2022).

Within the diverse categories of fermented foods, those primarily consisting of lactic acid bacteria (LAB) communities are of paramount significance and have been subjected to a full range of studies for quality improvement and mitigation of respective safety concerns (Wang et al., 2010, 2015; Hu et al., 2022). As illustrated by some studies, Beijing douzhi, also referred to as douzhir or douzhier, is a traditional snack rich in LAB. It originated in Beijing, China, and has been recognized as a fundamental element of Beijing’s intangible cultural heritage. This fermented delicacy is not only consumed by a substantial number of local inhabitants but also draws popularity among a broader audience, including tourists (Ding et al., 2009; Chen et al., 2013; Xu et al., 2018). This traditional fermented beverage is crafted through the natural fermentation of mung bean as its fundamental substrate (Huang et al., 2018). However, in contrast to its the extensive renown, the current production of douzhi is predominantly carried out by small-scale, artisanal techniques. The processing methods and utilization of starter cultures in its production are passed through successive generations of practitioners without adherence to industry-standard techniques, which may introduce variation in taste and result in noticeable disparities in product quality (Ding et al., 2010). Additionally, studies suggest that traditional spontaneous food fermentation, such as cereals, vegetables, and dairy, requires research efforts and appropriate measures to ensure safety and quality (Bleve et al., 2014; Perricone et al., 2014; Capozzi et al., 2017; Motato et al., 2017).

As a quintessential fermented food, comparable to yogurt and alcoholic beverages, the control of fermenting microorganisms and the addition of fermentation substrates are the critical factors that determine quality and safety in douzhi production (Sharma et al., 2020). While some research studies have been conducted to survey the general microbial communities and fermentation characteristics of douzhi, there is still a lack of in-depth interpretation regarding the microorganisms during its fermentation process (Ding et al., 2009; Chen et al., 2013). Also, given the absence of established and standardized fermentation procedure for douzhi, we hypothesized that by enhancing our comprehension of the dynamics of fermentation microbiota, we could improve the douzhi fermentation, thereby enhancing product quality and safety. Therefore, we designed the study with two main objectives: (A) supplementing additional beneficial LAB into the douzhi microbiota to enhance and modify the traditionally non-directional fermentation process, and (B) altering the traditional mung bean-based matrix to a non-native carbohydrate and protein-rich matrix (lactose and milk) to investigate the impact of two crucial factors in fermented foods—microbes and matrices—on douzhi fermentation. Hereafter, the findings from this study hold promise in advancing our understanding of the evolving role of fermentation control in dietary practices.

## 2 Materials and methods

### 2.1 Fermentation matrices preparation

Mung bean-based fermentation matrix was prepared using commercially available mung bean flour, finely grounded and filtered to achieve a particle size of less than 0.15 mm. This matrix was compared with a dairy-based matrix consisting of dehydrated skimmed milk (Difco™, Becton Dickinson, Detroit, MI, USA). In both matrix groups, 0.3 g of matrix powder was weighted and individually transferred into 10 mL screw-cap glass tubes. Sequentially, 10 mL deionized water was added, and the glass tubes were vigorously vortexed to ensure complete dissolution of soluble compounds. After vortexing, the sterilization process was performed by autoclaving the glass tubes at 121°C and 15 psi for 15 min.

### 2.2 Lactic acid bacterial inoculum

Cultures of *Pediococcus pentosaceus* CGMCC 1.2695 (isolated from dry beer yeast) and *Lactiplantibacillus plantarum* CGMCC 1.1856 (isolated from corn silage) were stored at −80°C, with the addition of 20% glycerol. To prepare precultures, the frozen bacterial strains were reactivated by incubating them in de Man, Rogosa, and Sharpe (MRS, Difco™, Becton Dickinson, Sparks, MD, USA) broth at 37°C for 18 h before being used in subsequent experiments. Inoculation was carried out by adding 100 μL of the revived MRS bacterial culture into 10 mL of fresh MRS broth, followed by an overnight incubation at 37°C. The bacterial cultures were then purified by centrifugation at 1811 × g for 5 min at 20°C and washing using sterile 0.85% NaCl solution. The optical density values of each resuspended culture were measured at 600 nm (OD_600_) and adjusted to a final OD_600_ value of 1.0 by adding sterile 0.85% NaCl solution as needed and temporarily stored at 4°C until inoculation. This yields an inoculum concentration of 9.02 ± 0.10 log CFU/ml for *Pediococcus pentosaceus* CGMCC 1.2695, and 8.85 ± 0.05 log CFU/ml for *Lactiplantibacillus plantarum* CGMCC 1.1856, as determined by plate counting.

### 2.3 Indigenous Beijing douzhi acquirement and fermentation initiation

In April 2023, douzhi samples were procured early in the morning, specifically prior to 8 a.m., from four local douzhi chain stores. The obtained samples were promptly chilled on ice and transported to the laboratory. The introduction of the douzhi microbial community into the fermentation matrices, signifying the initiation of the fermentation process (“time 0” of the fermentation), occurred immediately upon the arrival of the douzhi samples. The experiments were conducted exclusively within a laminar flow cabinet (DL-CJ-1NDII, HDL, Beijing, China) to minimize potential environmental contaminations. To ensure a uniform and balanced inoculation of the total douzhi microbial community, with particular attention to both the liquid and solid components, the douzhi samples were carefully transferred into sterilized and bakers with continuous agitation using a magnetic stirring plate. Subsequently, 250 μL of douzhi samples were transferred into each sterilized tubes that contains 10 mL of sterilized matrix (mung bean or skimmed milk). Following this, the inoculation with adjusted LAB suspension was performed immediately and mixed with rapid pipetting. This inoculation was carried out by adding 100 μL adjusted culture suspension (OD_600_ = 1.0) or 100 μL sterile 0.85% NaCl solution (for control purposes) into sterilized matrix tubes (10 mL), resulting in a 1:100 inoculation ratio, this resulting a. Aerobic fermentation was conducted under aseptic conditions without agitation utilizing a microbial incubator (HPS-400B, HDL, Beijing, China), with the tube caps positioned loosely to facilitating the exchange of air.

This experimental design comprised four distinct groups: mung bean matrices inoculated with douzhi microbiota without further inoculation (designated as the “control” or “C” group), mung bean matrices inoculated with douzhi microbiota and 6.86 ± 0.10 log CFU/mL *Pediococcus pentosaceus* CGMCC 1.2695 (designated as the “P” group), mung bean matrices inoculated with douzhi microbiota and 6.63 ± 0.14 log CFU/mL *Lactiplantibacillus plantarum* CGMCC 1.1856 (designated as the “L” group), and finally, skimmed milk matrices inoculated with douzhi microbiota without further inoculation (designated as the “M” group) (populations of LAB inoculum was enumerated with MRS plating method). More than 32 biological replications (inoculated matrix tubes) for each group were created at time 0 within a short timeframe (3 min). At each designated sampling time, a minimum of four tubes per experimental group were sampled and subjected to analyses. After conducting the necessary tests and sample retention, each sample was discarded, and new tubes were examined in the next sampling time point to ensure that the fermentation process proceeded without any intervening factors.

### 2.4 Microbial population and pH measurements

Samples from 4 local stores and fermented after 0, 2, 4, 8, 12, and 24 h were surveyed for microbial population evaluation and pH measurements. At each time point, 100 μl of homogenized fermentation cultures were made into a series of decimal dilutions using sterilized peptone saline solutions (comprising 0.1% peptone and 0.85% NaCl). Subsequently, population enumeration was conducted by duplicated plating of 100 μL of the dilutions on plate count agar (PCA, Difco™, Becton Dickinson, Sparks, MD, USA) and MRS agar, followed by an aerobic incubation at 37°C for 24 h. The pH values for each sample at each sampling time was measured by a pH meter (PB-10, Sartorius, Göttingen, Germany) at each sampling time.

### 2.5 Sample retention and DNA purification

Upon sampling and examination of 4 douzhi stores and experimental fermentation after 0, 4, 12, and 24 h, 1 mL of each tube was transferred to a clean 2-mL Eppendorf tube and immediately centrifuged at 16,099 × *g* for 2 min at 4°C. Supernatant was carefully removed then the pellet was rapidly frozen using liquid nitrogen and stored at −80°C until the DNA extraction was initiated. The purification of total DNA from the pellet was conducted using the TaKaRa MiniBEST Bacterial Genomic DNA Extraction Kit (Takara Ltd., Tokyo, Japan) in accordance with the manufacturer’s instructions. To determine the DNA concentration and purity, an assessment was performed with NanoDrop 2000 spectrophotometer (Thermo Fisher Scientific, Wilmington, DE, USA).

### 2.6 Amplicon library preparation and sequencing

Purified DNA samples were diluted to the same concentration (10 nmol/L) using 1 × Tris–EDTA buffer (pH 8.0). Then diluted samples were subjected to polymerase chain reaction (PCR) amplicon library construction with universal primer pairs (338F 5’-ACT CCT ACG GGA GGC AGC AG-3’ and 806R 5’-GGA CTA CHV GGG TWT CTA AT-3’) (Shanghai Shenggong Biotechnology Co., Ltd., Shanghai, China) targeting the V3-V4 hypervariable region of 16S rRNA gene and TransStart™ FastPfu Polymerase (TransGen Biotech Co., Beijing, China) in accordance with the manufacturer’s instructions. The PCR amplification was performed using ABI GeneAmp® 9,700 PCR System (Life Technologies, Foster City, CA). The PCR program setting was 3 min at 95°C Followed by 30 cycles of 30 s at 95°C, 30 s at 55°C, 30 s at 72°C. And a final elongation (10 min at 72°C, and 4°C until sample collection) was added at the end of the PCR run. Amplicon products were evaluated using agarose gel electrophoresis. High-throughput sequencing of amplicon products was performed on the Illumina NovaSeq 6,000 system (Illumina, San Diego, CA, USA).

### 2.7 Quality control and data processing

The sequencing process produced paired-end sequences, 250 base pairs (bp) from each side. in FASTQ format for each amplicon product. Subsequently, Cutadapt (version 3.4) was employed to remove the attached barcodes and primers from both ends of the amplicons (Martin, 2011). To further refine the dataset, FastQC (version 0.12.1) was utilized to filter out sequences with low quality (defined as those with an initial quality score lower than 31) (Andrews, 2010). Finally, paired-end sequences were merged according to overlapping area using FLASH (version 1.2.11) (Magoč and Salzberg, 2011). Merged sequences were traded as single-end sequences and investigated using QIIME 2 pipeline (version 2023.2) (Bolyen et al., 2019). The DADA2 package (Callahan et al., 2016) was utilized to generate amplicon sequence variant (ASV) tables and filter chimeric sequences. These tables were curated to represent ASVs with an average length of 120 bp targeting the V4 hypervariable region.

### 2.8 Microbial community analyses

Based on rarefaction analysis, a unified sampling depth of 30,000 was selected for all subsequent analyses to ensure uniformity and accuracy. The taxonomic classification for each sample was determined by assigning ASV tables to reference sequences of weighted full-length SILVA database (release 138) (Quast et al., 2012; Kaehler et al., 2019). This assignment was accomplished using a Naive Bayes classifier (Wang et al., 2007). The relative abundance of specific taxonomic classifications was calculated as the percentage of ASV numbers, while excluding chloroplast and mitochondrial ASVs from the analysis. Alpha diversity of samples was calculated based on Faith’s phylogenetic diversity (PD) index (Faith, 1992; Thukral, 2017), Shannon’s diversity index (Keylock, 2005), or Pielou’s Evenness index (Pielou, 1966). Subsequently, principal coordinate analysis (PCoA) plots for beta diversity analysis were generated using the q2-diversity plugin and visualized through Emperor (Vázquez-Baeza et al., 2013). The PCoA plots in this study were plotted based on the Bray–Curtis’ dissimilarity matrix, which quantifies ASV differences.

### 2.9 Microbial community function prediction

To predict the functions of each microbial communities, ASV tables were subjected to further analysis using PICRUSt2 (Douglas et al., 2020). This analysis was carried out with phylogenetic placement tool EPA-ng, and function hidden-state prediction based the maximum parsimony principles (Kolaczkowski and Thornton, 2004; Zaneveld and Thurber, 2014; Barbera et al., 2019). Output results of each sample include MetaCyc pathways and enzyme commission (EC) numbers. To further elucidate the functional significance, enrichment analysis of EC functions was conducted employing the KEGG mapper (Bairoch, 2000; Kanehisa and Goto, 2000; Caspi et al., 2006).

### 2.10 Metabolomic profiling of fermentation supernatant via liquid chromatography–mass spectrometry analysis

Metabolomic analysis based on liquid chromatography–mass spectrometry is carried out to elucidate the alterations in chemical and nutritional characteristics introduced by microbial fermentation. Four experimental groups were subjected to analysis in this section: C0: consisting of mung bean matrices inoculated with douzhi microbiota without fermentation; C24: comprising mung bean matrices inoculated with douzhi microbiota and fermented for 24 h; P24: mung bean matrices were inoculated with douzhi microbiota and *Pediococcus pentosaceus* CGMCC 1.2695, then fermented for 24 h; L24: mung bean matrices were inoculated with douzhi microbiota and *Lactiplantibacillus plantarum* CGMCC 1.1856, followed by 24 h of fermentation.

For each sampling time, six samples from each group were quickly transferred into sterilized beakers, at which continuous agitation was ensured via magnetic stirring. Subsequently, 1 mL of homogenized fermentation matrix was transferred to clean 2-mL Eppendorf tubes and centrifuged at 16,099 × g for 2 min at 4°C. A 200 μL aliquot of the supernatant from each sample was carefully transferred into individual 1.5 mL centrifuge tubes and mixed with 800 μL of the extraction solution. The extraction solution consisted of methanol and acetonitrile in a ratio of 1:1 (v/v) and contained four internal standards. After ultrasonic extraction, incubation, and centrifugation, supernatants were transferred and dried using nitrogen. The dried residue was reconstituted with 120 μL of reconstitution solution (acetonitrile: water = 1:1) and subjected to further ultrasonic extraction. Eventually, supernatants were transferred to sample vials for analysis, while 20 μL of supernatant from each sample was mixed and analyzed for quality control (QC) purposes. Ultrahigh-performance liquid chromatography coupled with the Q Exactive™ HF-X Hybrid Quadrupole-Orbitrap™ mass spectrometer (Thermo Fisher Scientific, Waltham, MA, USA) was employed to analyze the metabolomic composition. Chromatographic separation was achieved using an ACQUITY UPLC HSS T3 column (Waters corporation, Milford, CT, USA) with a mobile phase consisting of 95% water and 5% acetonitrile as phase A and 47.5% acetonitrile, 47.5% isopropanol, and 5% water as phase B. For mass spectrometry, parameters were set as follows: mass range m/z of 70–1,050, sheath gas flow rate of 50 arbitrary unit (arb), auxiliary gas flow rate of 13 arb, heater temperature of 425°C, capillary temperature of 325°C, spray voltage of 3,500 V in positive ion mode and −3,500 V in negative ion mode, normalized collision energy of 20, 40, and 60%, full MS resolution of 60,000, and MS/MS resolution of 7,500. The liquid chromatography–mass spectrometry data obtained was subjected to analysis using Progenesis QI (v3.0, Waters corporation, Milford, CT, USA) for peak picking, retention time alignment, compound annotation and subsequential analysis, while metabolites were identified according to Human Metabolome Database (HMDB)^1^. Differential abundance analysis was carried out employing the relative betweenness centrality. The Pearson correlation coefficient (r) was calculated in R (version 4.4.0) to assess the cross-correlation between dominant taxonomic groups and metabolites using the psych package, while heatmap was visualized with the pheatmap package.

### 2.11 Statistical analysis

Statistical analyses were performed using SPSS software (SPSS Statistics for Windows 21.0.0; SPSS Inc., Chicago, IL, USA). A minimum of three biological replicates were examined for each experiment. The counts of culturable microbial populations, as determined by the plate counting method, were transformed into their logarithmic form for analyses. Single-factor analysis of variance (ANOVA) was employed to assess group differences, and statistical significance (*p* < 0.05) was determined using Tukey’s range test to calculate *p*-values. Data normality was assessed through the Kolmogorov–Smirnov test, followed by either Student’s *t*-test or the Mann–Whitney U test for comparing two groups, as applicable, while in differential abundance analysis and cross-correlation analysis, Benjamini-Hochberg false discovery rate (FDR) controlling procedure was utilized to calculate the FDR adjusted *p-*value. Statistical significance levels were denoted as follows, ns (not significant): *p* > 0.05; *p < 0.05; **p < 0.01; ***p < 0.001; ****p < 0.0001.

## 3 Results

### 3.1 Microbial population and pH of indigenous douzhi

**Figure 1:**
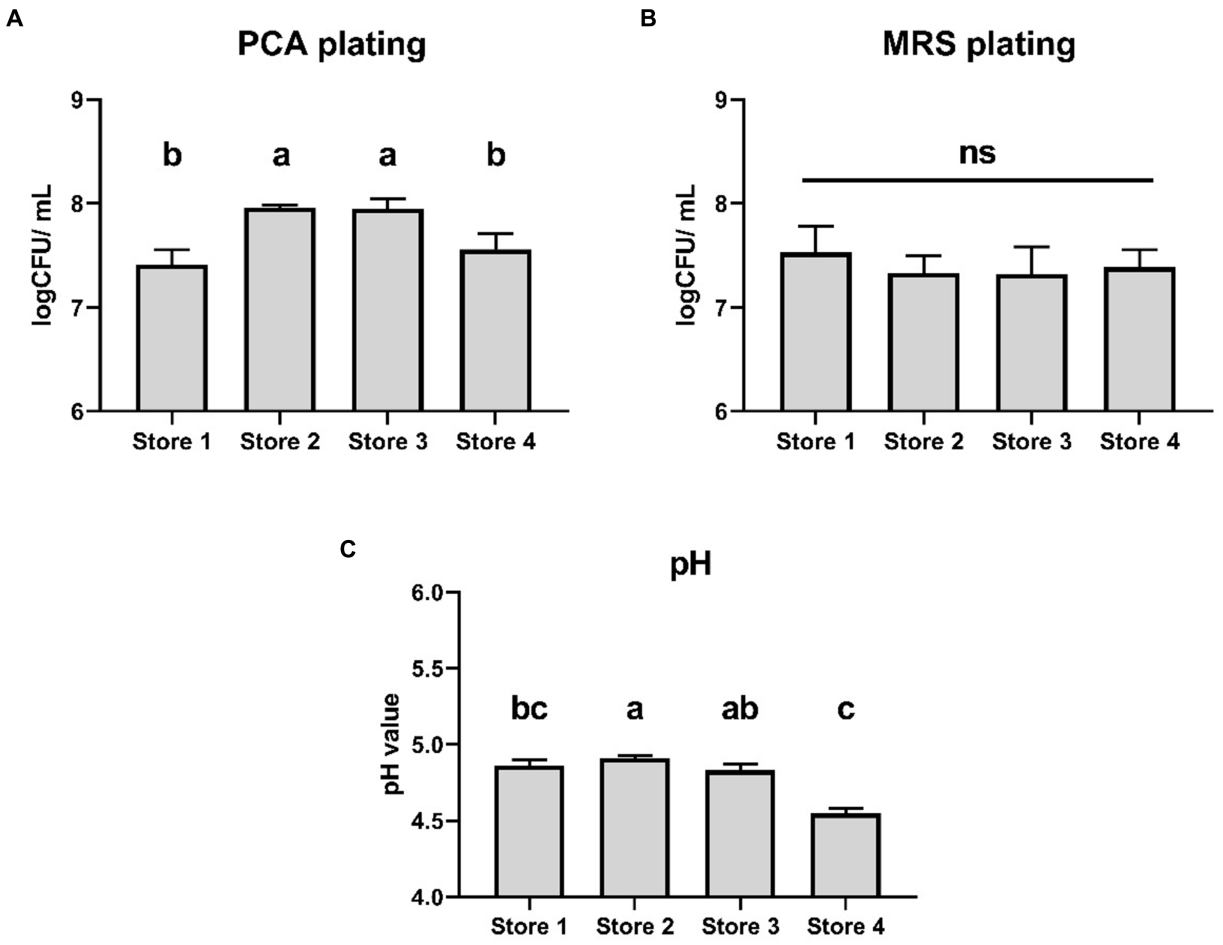
Microbial population and pH of indigenous douzhi freshly purchased from 4 local stores. **(A)** Aerobic population measured using PCA agar plating. **(B)** Microbial population measured using MRS agar plating. **(C)** The pH value. Data are expressed as the mean ± standard deviation (SD), *n* = 3. Different capitalized letters at different groups indicate significant differences (*p* < 0.05), as determined by the Tukey’s range test.

The four douzhi stores selected for the experiment are retail outlets operating under a larger corporation, and they are in close proximity to the laboratory. After purchasing, the douzhi samples were chilled on ice and delivered to the laboratory within 20 min, then immediately subjected to sampling and measurements. The data revealed that the total bacterial count recorded by PCA agar plates was slightly lower in Store 1 (7.41 ± 0.15 log CFU/ml) and Store 4 (7.56 ± 0.15 log CFU/ml) compared to store 2 (7.96 ± 0.03 log CFU/ml) and store 3 (7.95 ± 0.10 log CFU/ml) (*p* < 0.05) (Figure 1A). However, there were no significant differences in the microbial population as measured by MRS agar plates among the four stores (Figure 1B). Additionally, the pH level in Store 4 (4.55 ± 0.93 log CFU/ml) was slightly lower than that of the other three stores (*p* < 0.05) (Figure 1C).

### 3.2 Amplicon sequencing quality assessment and indigenous douzhi microbiota

In this study, paired-end sequencing was performed on samples from both the douzhi store and fermentation processes, utilizing primer sets that yielded final PCR amplicons with an average size of 469 bp (Supplementary Figure S1A). Despite variations in DNA concentration and purity across different samples (Supplementary Figures S2A,B), the amplicon products and sequential high-throughput sequencing exhibited high quality, as indicated by the gel electrophoresis visualizations (Supplementary Figure S3), presence of *Q*30 scores (Supplementary Figure S1B) (the percentage of total sequences with quality scores equal to or exceeding 30, which corresponds to a precision level of 99.9%), as well as high overall sequencing depths (Supplementary Figure S1C). These indicators are crucial in ensuring the reliability of the sequencing data, which showcased that sample handling procedures and high-throughput sequencing approaches can robustly assesses microbial communities in douzhi and fermentation experiments.

**Figure 2:**
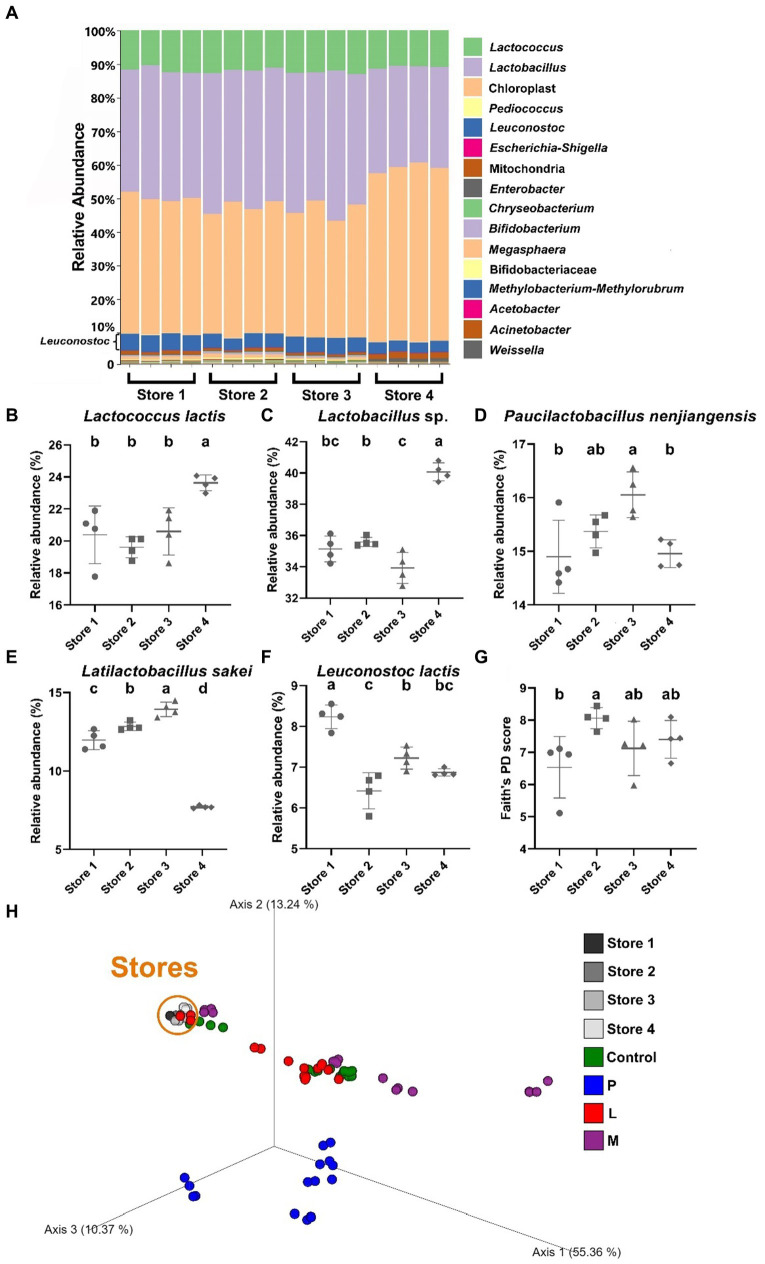
Taxonomic and phylogenetic analyses of 4 douzhi stores. **(A)** Stacked bar chart showing the taxonomic composition of samples at the genus level. The taxonomic classification order (top to bottom) in the legend corresponds to that in the figure. **(B–F)** Relative abundance of key species. **(B)** *Lactococcus lactis,* **(C)** *Lactobacillus* sp., **(D)** *Paucilactobacillus nenjiangensis,* **(E)** *Latilactobacillus sakei,* **(F)** *Leuconostoc lactis,* **(G)** Alpha diversity assessed using Faith’s PD index. **(H)** PCoA conducted using the Bray–Curtis dissimilarity matrix encompassing all 80 samples in this study. Data are expressed as the mean ± SD, *n* = 4. Different capitalized letters at different groups indicate significant differences (*p* < 0.05), as determined by the Tukey’s range test.

By employing QIIME 2 and the DADA2 pipeline, tables of amplicon sequence samples with an average sequence length of 120 base pairs (nucleotides 570–689, theoretically) were generated, effectively covering the entire 16S rRNA V4 hypervariable region (nucleotides 576–682) (Chakravorty et al., 2007). Then, taxonomic classification was determined for all samples through alignment against the weighted SILVA database. At the genus level, the analysis of stacked taxonomic bar charts provides compelling evidence for the existence of well-established and thriving microbial communities that were abundant in LAB across all four stores (Figure 2A). The predominant constituents within these communities primarily belong to the Lactobacillaceae family and the genus *Lactococcus*, collectively accounting for more than 90% of the total ASVs when excluding chloroplast and mitochondria sequences. Microbial species composition differences were observed in different stores. Specifically, in Store 4, we found a higher relative abundance of *Lactococcus lactis* and Lactobacillaceae when compared to the other three stores (*p* < 0.05) (Figures 2B,C). On the other hand, Store 3 exhibited a higher relative abundance of *Paucilactobacillus nenjiangensis* and *Latilactobacillus sakei* (*p* < 0.05) (Figures 2D,E), while Store 1 had a higher relative abundance of *Leuconostoc lactis* (*p* < 0.05) (Figure 2F) compared to the other stores.

Moving on, assessments of alpha diversity were conducted, aiming to evaluate sample phylogenetic coverage and species richness while disregarding specific taxonomic distinctions among groups. The results revealed no statistically significant differences in alpha diversity among Store 2, Store 3, and Store 4. However, Store 1 exhibited significantly lower alpha diversity (6.54 ± 0.83) (*p* < 0.05) only compared to Store 2 (8.06 ± 0.28), as indicated by Faith’s PD index (Figure 2G). Beta diversity analysis, utilizing the Bray–Curtis dissimilarity matrix, was performed on samples collected from the four stores as well as all the samples from the fermentation experiments. This analysis was designed to distinguish specific taxonomic differences among all the samples. The objective was to illustrate the changes in overall diversity, which were visualized on PCoA plots (Figure 2H). In these plots, the top three principal coordinates were employed as the set of orthogonal axes. Remarkably, clear differences were evident in the distances among the fermentation samples, with the predominant variation occurring along axis 1 which contributed to approximately 55.36% of the total variability. Remarkably, all samples from the douzhi store exhibited close clustering within a relatively confined area, particularly when compared with the microbial communities from the fermented samples. Store 4 was arbitrarily chosen as the source of the fermentation starter for LAB and the matrix fermentation experiments. This selection would provide sufficient representation for the chain stores, particularly considering the relatively minor distinctions among the different store locations.

### 3.3 Microbial population and pH changes in fermentation experiments

**Figure 3:**
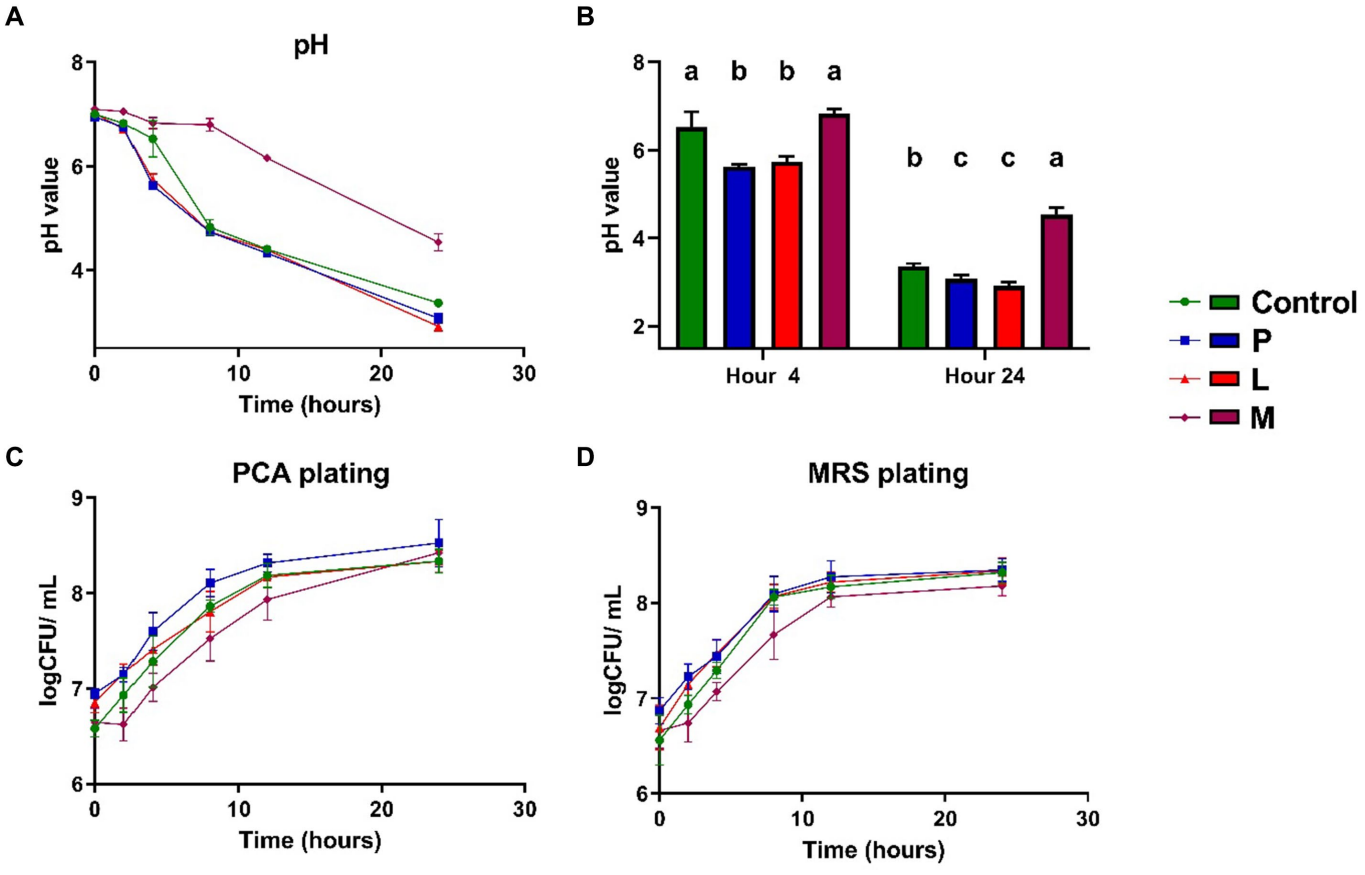
Microbial population and pH changes throughout the 24-h fermentation period. **(A)** The pH value. **(B)** Examination of pH values at the 4-h and 24-h time points. **(C)** Aerobic population measured using PCA agar plating. **(D)** Microbial population measured using MRS agar plating. Data are expressed as the mean ± SD, *n* = 3. Different capitalized letters at different groups with in a time point indicate significant differences (*p* < 0.05), as determined by the Tukey’s range test.

The pH and culturable microbial population of the group C, P, L, and M were analyzed using conventional laboratory techniques. At the outset, all four groups exhibited a pH value in close proximity to neutrality (6.95 to 7.1), with no significant differences (*p* > 0.05) among them (Figure 3A). As the fermentation process initiated, both group P and group L experienced a substantial reduction in pH within the first 4 h. Notably, their pH values (5.63 ± 0.05 for group P, 5.74 ± 0.11 for group L) were significantly lower (*p* < 0.05) than those of the group C (6.53 ± 0.35) and group M (6.83 ± 0.10) at the 4-h time point (Figure 3B). Throughout the subsequent stages of fermentation, beyond the 4-h mark, the pH of the control group decreased more rapidly, approaching the levels of the P and L groups. However, in the long term (after 24 h), both the group P (3.07 ± 0.10) and group L (2.92 ± 0.10) exhibited a slightly lower pH value (*p* < 0.05) in comparison to the control group (3.37 ± 0.06) (Figures 3A,B). In the M group, although the pH exhibited a significant decrease (from 7.09 ± 0.18 to 4.54 ± 0.16), the rate of pH decreases was lower and the absolute pH values at various time points were notably higher (*p* < 0.05) in the M group compared to the other three mung bean-based fermentation matrices (Figures 3A,B). As for microbial population detected by plate count method, no noticeable population differences were observed among total aerobic populations (Figure 3C) and microbial populations measured by MRS (Figure 3D), especially at end of the fermentation (*p* > 0.05), as both the total aerobic microbial population and the LAB population exhibited a gradual and similar increase throughout the course of fermentation. Additionally, all three groups utilizing mung bean-based matrices displayed an apparent increase in turbidity (Supplementary Figure S4).

### 3.4 Taxonomic and phylogenetic dynamics of fermented douzhi microbiota

**Figure 4:**
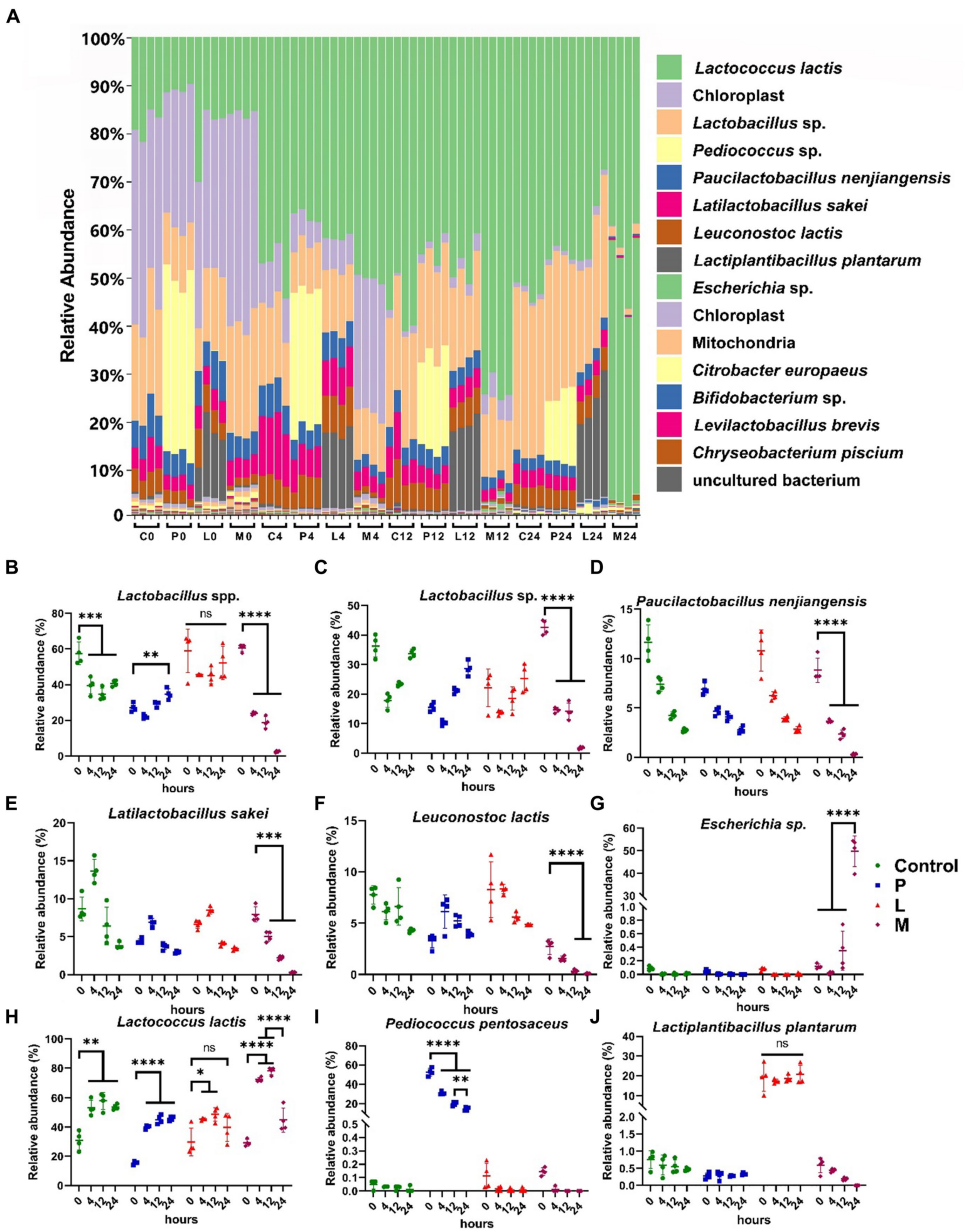
Taxonomic classification dynamics during the 24-h fermentation. **(A)** Stacked bar chart showing the taxonomic composition of samples at the species level. Samples are denoted as “group (C, P, L, and M) and fermentation time (hour) (0, 4, 12, 24).” The taxonomic classification order (top to bottom) in the legend corresponds to that in the figure. **(B)** Relative abundance of *Lactobacillus* spp. **(C–J)** Relative abundance of key species. **(C)** *Lactobacillus* sp., **(D)** *Paucilactobacillus nenjiangensis,* **(E)** *Latilactobacillus sakei,* **(F)** *Leuconostoc lactis,* **(G)** *Escherichia* sp., **(H)** *Lactococcus lactis,* **(I)** *Pediococcus pentosaceus,* **(J)** *Lactiplantibacillus plantarum*. Data are expressed as the mean ± SD, *n* = 4. Differences are determined by Student’s *t*-test, ns (not significant): *p* > 0.05; *p < 0.05; **p < 0.01; ***p < 0.001; ****p < 0.0001.

Using the same amplicon sequencing pipelines as adopted by douzhi store analysis, we conducted a comprehensive taxonomic and phylogenetic analysis of a total of 64 samples across four experiment groups in the fermentation study. In the detailed stacked taxonomic bar chart at species level, it becomes apparent that the majority of the samples exhibit a continuous decrease in the proportion of chloroplast ASV counts. This trend coincided with the continual proliferation of microorganisms throughout the entire fermentation. Furthermore, it is noteworthy that the microbial community structure in the majority of samples demonstrates a sustained predominance by the LAB species, with the sole exception found in the 24-h fermented M group (Figure 4A). In particular, the relative abundances of some Lactobacillaceae (identified as *Lactobacillus* spp. in classification) and *Leuconostoc lactis* displayed comparable abundance dynamics among the four groups and usually stay at an elevated level, while their significant and dramatic decreases were observed during the fermentation in group M (Figures 4B–F). This reduction was particularly pronounced at the 24-h mark, where their abundance plummeted to extremely low levels. Specifically, the abundance of some Lactobacillaceae (identified as *Lactobacillus* spp. in classification) decreased significantly from 60.61% ± 1.73% to a mere 2.43% ± 0.51% (*p* < 0.001) (Figure 4B). This observation finds further support through the species-level classification of Lactobacillaceae (Figures 4C–E). *Leuconostoc lactis* also experienced a substantial reduction, declining from 2.43% ± 0.51 to 0.09% ± 0.04% (*p* < 0.001) during the same fermentation period in group M (Figure 4F). Concurrently, the relative abundance of *Escherichia* sp. dramatically increased from below 1% to a remarkably 49.74% ± 5.93% (Figure 4G). It is intriguing to note that *Lactococcus lactis*, another prominent constituent of LAB, exhibited notably distinct dynamics. After 12 h of fermentation, its abundance significantly increased across all four groups (*p* < 0.05), and this increase was sustained in both group C (*p* < 0.01) and group P (*p* < 0.001) at hour 24 when compared to hour 0 (Figure 4H). While the abundance of *Lactococcus lactis* did decrease in group M at hour 24 when compared to hour 12 (*p* < 0.001), it still maintained a relatively high level (44.92% ± 7.24%). This distinct behavior sets it apart from the other LAB components, highlighting its exceptional resilience in facing the detrimental stress experienced by LAB in group M.

**Figure 5:**
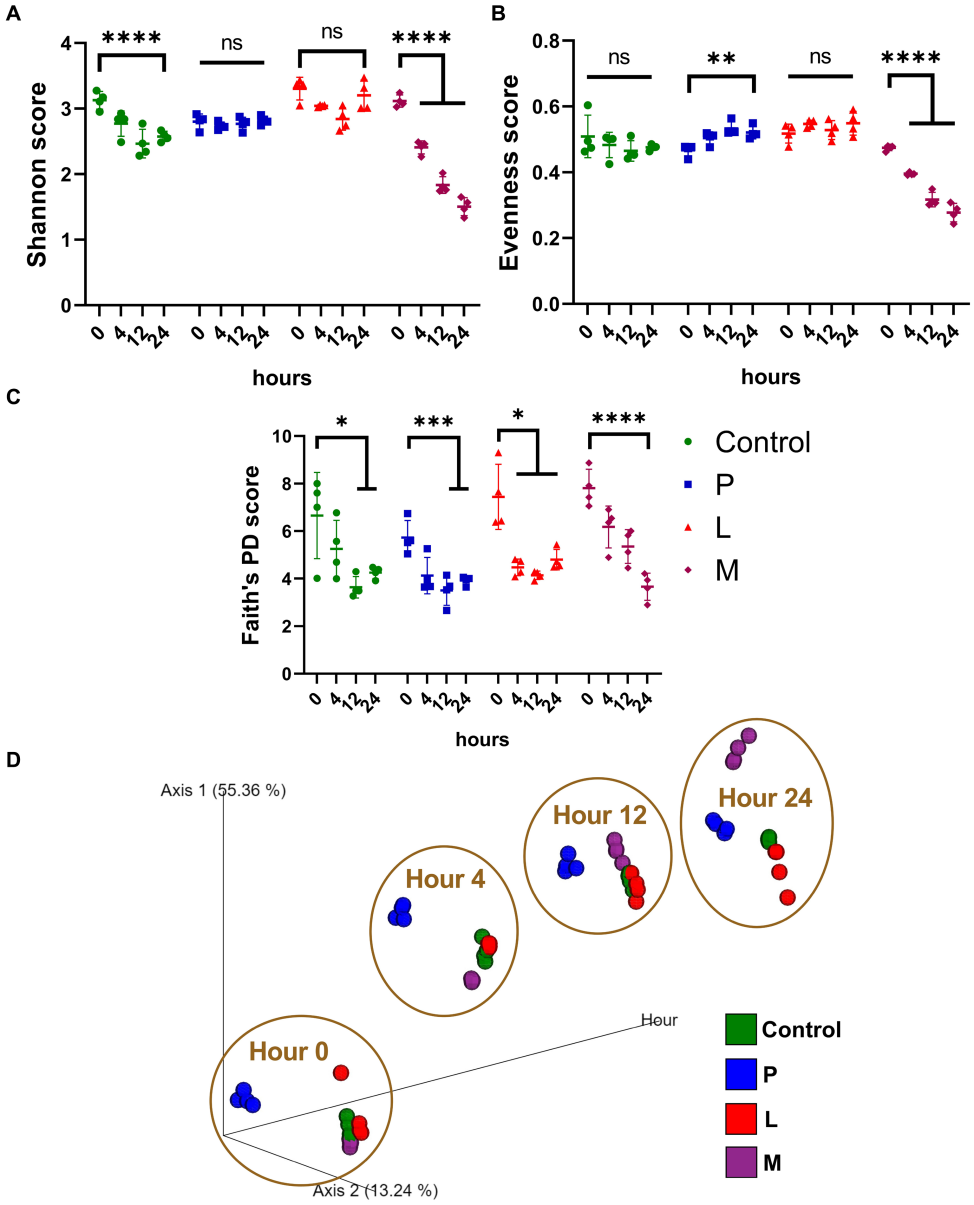
Phylogenetic dynamics during the 24-h fermentation. **(A–C)** Alpha diversity assessed using different mythologies. **(A)** Shannon’s diversity index **(B)** Pielou’s evenness index **(C)** Faith’s PD index **(D)** PCoA was performed using the Bray–Curtis dissimilarity matrix, originated on the top two coordinates (Axis 1 and Axis 2) and fermentation hours as Axis 3.

Alpha diversity was assessed using methodologies such as Shannon’s diversity index, and the results revealed a significant decrease (*p* < 0.001) in Shannon score, from 3.13 ± 0.01 (hour 0) to 2.58 ± 0.07 (hour 24) within the control group and a much more pronounced decrease (*p* < 0.001) in group M when comparing the samples collected at hour 0 (3.11 ± 0.08) and hour 24 (1.51 ± 0.12) within a group (Figure 5A). In contrast, no significant change (*p* > 0.05) in alpha diversity was observed within both group P and group L over the same time period. When applying Pielou’s evenness index, we observed similar dynamic patterns, most prominent decrease (*p* < 0.001) was observed in group M, with values changing from 0.47 ± 0.01 at hour 0 to 0.28 ± 0.02 at hour 24 (Figure 5B). When examining Faith’s PD index, statistically significant decreases (*p* < 0.05) were observed in all four groups over the 24-h fermentation period (Figure 5C). The measurement in Faith’s PD index exhibited substantial dependency on the extent of phylogenetic coverage and species richness, while the Shannon’s diversity index and Pielou’s evenness index predominantly emphasized on species evenness (Pielou, 1966; Faith, 1992; Keylock, 2005; Thukral, 2017). The beta diversity analysis, employing the consistent Bray–Curtis dissimilarity matrix method as discussed in the previous section (Figure 5D), utilized the top two principal coordinates along with fermentation hours as orthogonal axes. The results revealed a distinct separation between group P and the other three groups throughout the entire fermentation period. Conversely, group M initially exhibited proximity to the control and group L but diverged significantly at the 24-h mark. Group L and the control group maintained close proximity to each other throughout the fermentation process.

### 3.5 Metagenomic functional predictions

**Figure 6:**
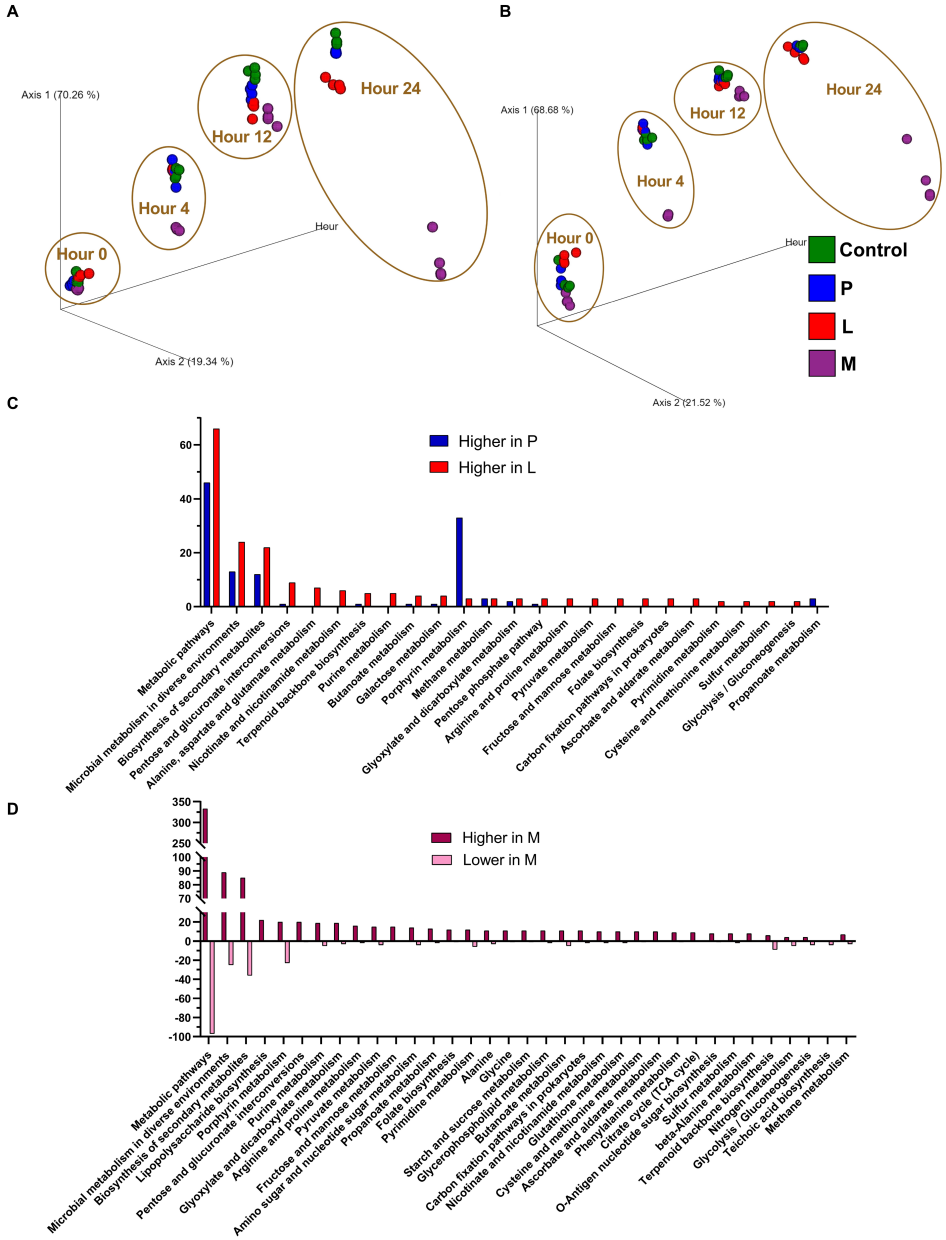
Microbial community function predictions by PICRUSt2. PCoA was performed using the Bray–Curtis dissimilarity matrix of **(A)** MetaCyc pathways and **(B)** enzyme commission (EC) numbers, which are originated on the top two coordinates (Axis 1 and Axis 2) and fermentation hours as Axis 3. **(C)** Enrichment analysis of ECs that are predicted to exhibit significantly elevated activity for both group P and group L, respectively, in comparison to the control group. **(D)** Enrichment analysis of ECs that are significantly changed for group M. ECs with an average relative abundance exceeding 250 in both the target group and the control, while the mean relative abundance in the target group is at least 150% higher or 66.7% lower (*p* < 0.0001) when compared with the control group, are selected for the enrichment.

In this study, hidden-state prediction methodology was employed by using PICRUSt2 to predict the comprehensive functional profile of the microbiome. PCoA plots, much like phylogenetic analyses, were generated to visually represent the Bray–Curtis dissimilarity matrix corresponding to the abundances of MetaCyc pathways (Figure 6A) and EC numbers (Figure 6B), originating from the results of four groups participating in the fermentation experiments. Both PCoA plot demonstrated that control, group P, and group M, characterized as LAB-dominated communities, exhibited close proximity throughout the entire fermentation process. However, a noteworthy difference occurred in group M, which employed a skimmed milk-based fermentation matrix. After 24 h of fermentation, it experienced a significant shift, with *Escherichia* sp. and *Lactococcus lactis* dominating the microbiota. As a result, it exhibited a marked departure from the other three mung bean-based matrices in metagenomic functions.

When the respective discrimination threshold for the counts reflecting the EC functions of the four groups was established, a noteworthy observation emerged. It was intriguing that when individually compared to the control group, neither group P nor group L exhibited lower enrichment of EC functions (Figure 6C). Contrastingly, both group P and group L exhibited notable increases in the number of enriched EC functions. Specifically, several functions, including metabolic pathways, microbial metabolism in diverse environments, and biosynthesis of secondary metabolites, exhibited an increase in activity in both group P and group L. Whereas certain functions, such as pentose and glucuronate interconversion and alanine, aspartate, and glutamate metabolism, experienced a distinct increase exclusively within group L. Furthermore, porphyrin metabolism displayed a remarkable surge in activity within group P, yet porphyrin metabolism increase was not observed in group L. This phenomenon could potentially be attributed to the introduction of newly inoculated microorganisms in both group P and group L, which may have introduced additional gene functions without significantly perturbing the original microbiota functions. In the case of group M, the situation is much different. Compared with control group, there were significant variations (both higher and lower) in the representation of enriched EC functions (Figure 6D). It can be inferred that within group M, a predominance of enriched EC functions exhibited a higher presence rather than a lower. This observation could be attributed to the transition in the microbiota composition of group M, shifting from a predominance of LAB to a co-dominance of *Lactococcus lactis* and *Escherichia* sp., both of which are highly represented in the microbiota at the 24-h mark (Figure 4).

### 3.6 Metabolomic analysis of fermented mung bean matrix

**Figure 7:**
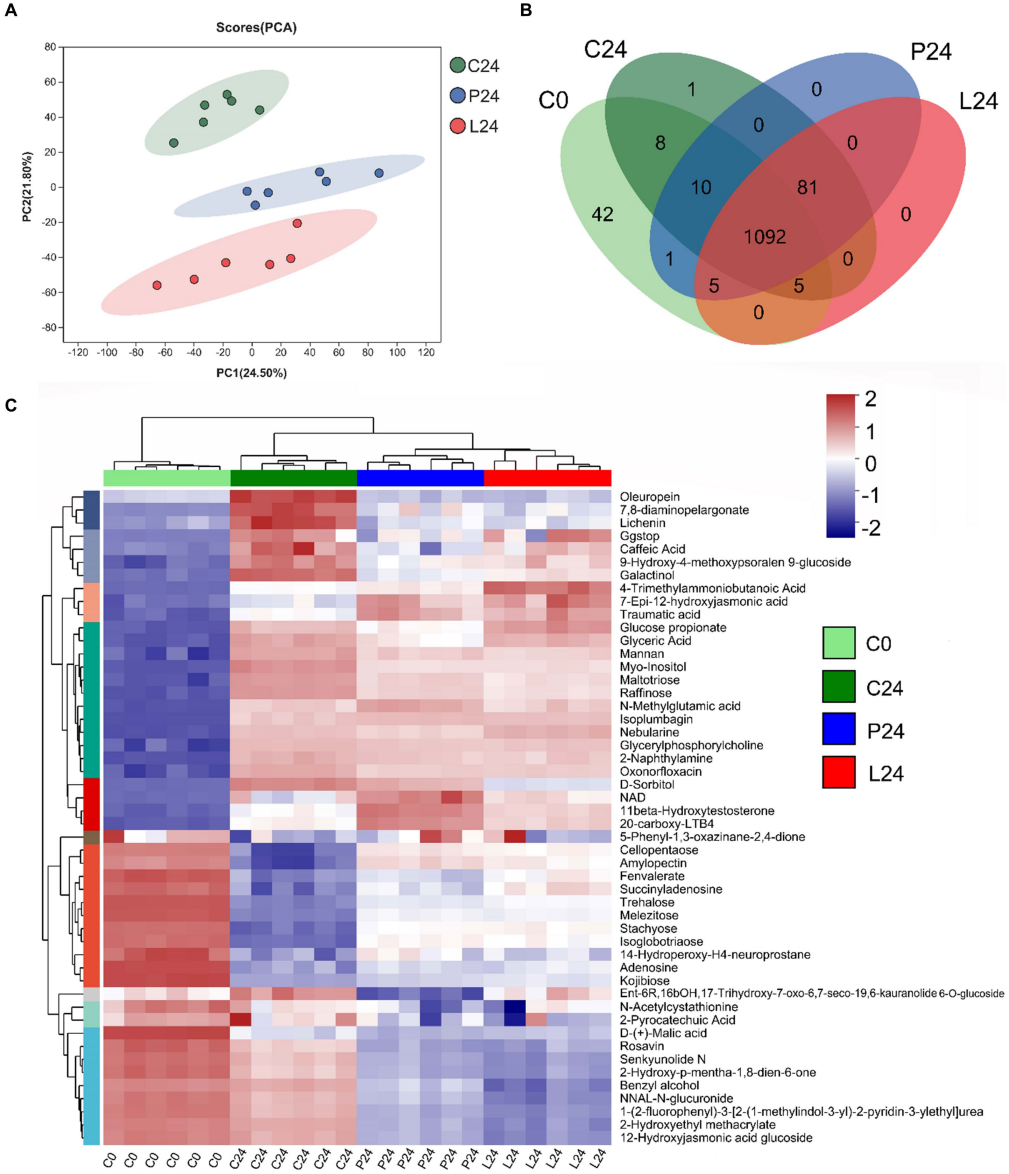
Metabolomic profile of fermented mung bean matrices with different starting microbiotas. **(A)** Principal component analysis (normalized with unit variance) of group C, P, and L after 24 h of fermentation. **(B)** Venn diagram illustrating the distribution of 1,245 metabolites among different groups, as detected by liquid chromatography–mass spectrometry. The numbers within each group area represent the metabolites positively identified. **(C)** Heat map representation of significant metabolites identified through correlation analysis. The color gradient, ranging from blue to red, indicates increasing concentrations of the specified metabolites.

**Figure 8:**
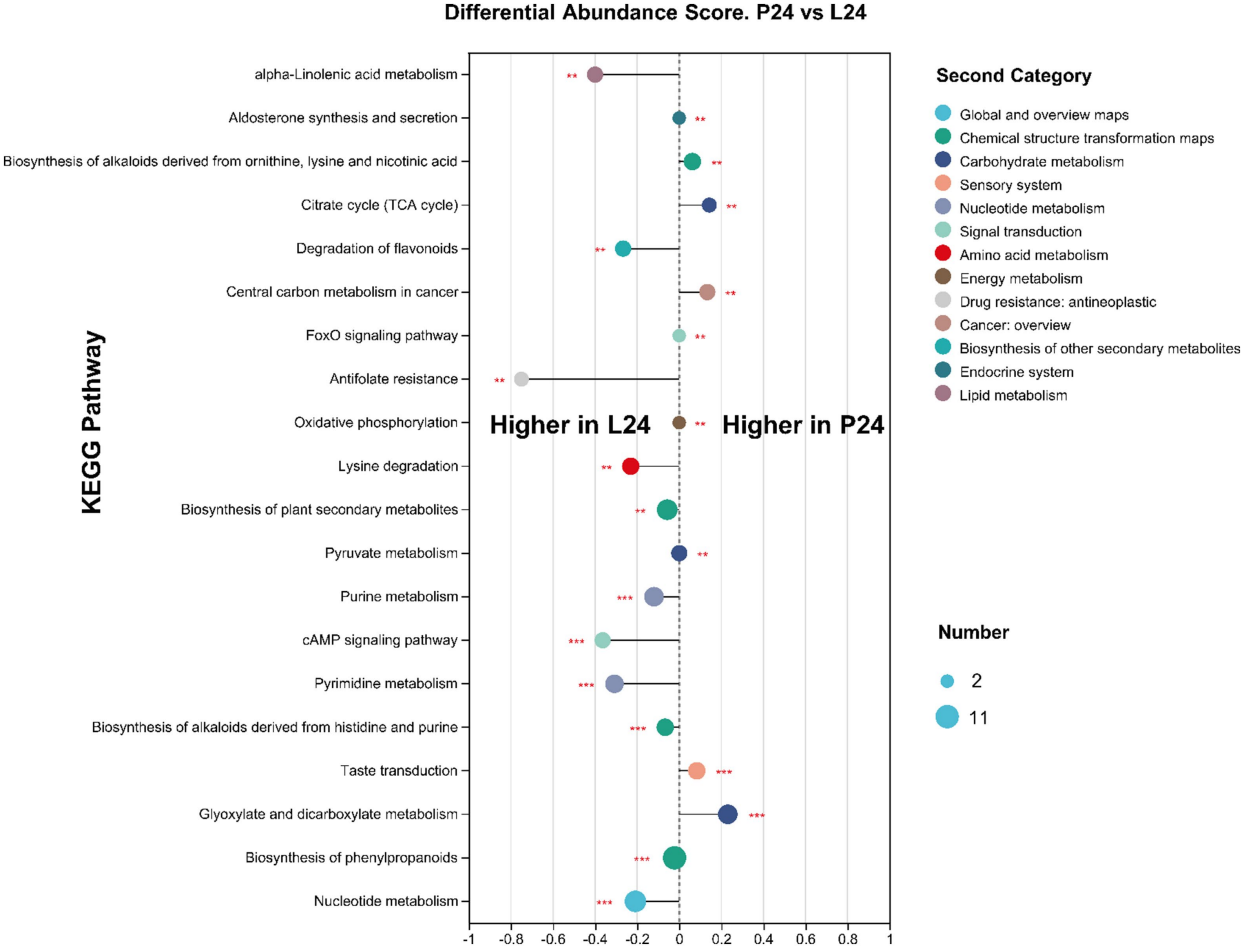
Differential abundance analysis between group P24 and L24 based on KEGG pathway enrichments of differential metabolites. A DA Score above zero suggests that a pathway is higher expressed in the P24 group, whereas a score below zero indicates higher expression in the L24 group. The circle size represents the number of differential metabolites annotated within the pathway. Differences are determined by FDR adjusted *p*-values, *n* = 6, **p < 0.01; ***p < 0.001.

In this study, we employed liquid chromatography–mass spectrometry to perform a comprehensive metabolomic analysis of the mung bean matrix before and after microbial fermentation (Detailed information can be found at metabolomic profile summary table, Supplementary Table S1). As expected, C24, P24, and L24 resulted in significant changes in metabolite profile. The addition of *Pediococcus pentosaceus* and *Lactiplantibacillus plantarum*, compared to douzhi community alone, produced apparent separation in principal component analysis (Figure 7A). It can be inferred from the Venn diagram that the fermentation of inoculated LAB resulted no new metabolite detected compared to douzhi community fermentation alone (Figure 7B). This observation suggests that the metabolic contributions of the added LAB overlap significantly with those of the indigenous douzhi microbial community. The metabolite concentration heatmap across the four investigated groups shows that P24 and L24 exhibited similar patterns, in contrast to C0 and C24 (Figure 7C). Particularly, higher concentration of low molecular weight carbohydrates, such as trehalose, melezitose, stachyose, and isoglobotriaose, was observed in both P24 and L24. Numerous other nutritionally significant substances, such as galactinol, D-sorbitol, and caffeic acid, displayed clear differences in concentration in P24 and L24, compared with C24 (Figure 7C). Metabolite pathway enrichment and associated differential abundance analysis serves as a powerful method for uncovering the underlying biochemical mechanisms behind the metabolic changes observed in the samples. The differential analysis revealed a higher degree and more significantly represented metabolic pathways in the L24, compared to P24 (Figure 8). This observation is consistent with metagenomic results, suggesting that the larger genome of *Lactiplantibacillus plantarum* may confer greater capacity to modulate the metabolic functions of the fermentation microbiota. Meanwhile, the cross-correlation analysis between the most abundant microbial species and metabolites indicates that *Lactiplantibacillus plantarum* CGMCC 1.1856 and *Lactococcus lactis* exhibited a negative correlation with their impact on key metabolites such as succinic acid, FAPy-adenine, and isocitric acid (Supplementary Figure S5). Similarly, *Pediococcus pentosaceus* CGMCC 1.2695 and *Latilactobacillus sakei* also showed a negative correlation with their impact on metabolites such as 1-Butylamine and 3-Aminopentanedioic Acid. These findings highlighted the competitive impact of the added LAB on the ecological niche and functionality of the indigenous strains.

## 4 Discussion

As demonstrated by previous studies, prokaryotic microorganisms, particularly LAB such as *Lactococcus* and Lactobacillaceae, assumed predominant roles in the fermentation process and the microbial communities of douzhi (Chen et al., 2013; Xu et al., 2018). These microorganisms were responsible for acid production and flavor enhancement. While eukaryotic microorganisms, specifically yeasts, can only exhibit significant proliferation in the latter phases of fermentation or during prolonged storage, their role is found to be associated with decrease in acidity (pH increases) which is generally undesirable in acidic fermented foods (Ding et al., 2009; Swain et al., 2014). In this study, we selected four douzhi stores that are retail outlets that operate under a broader corporate framework. These choices were made to not only illustrate large scale douzhi production but also to exemplify a microbiome with a certain level of representativeness, ensuring that the obtained microbiome is both representative and of significant research value. While there are slight variations in the aerobic microbial population and pH levels (Figure 1), overall, the primary parameters of these douzhi products are relatively similar. The chain store setting of these stores implies a consistent overall production process, though variations may occur due to differences in storage, transportation, retail, and serving conditions at each location. It should be pointed out that traditional douzhi products do not exhibit uniform stability, rather, they can be regarded as suspensions containing small solid particles that gradually precipitate as the static settling time increases. As a result, there is a notable variation in the quantification of viable bacterial counts in douzhi samples collected at different settling times and from various areas within the larger settling container, leading to discrepancies in culturable population counts.

To comprehensive elucidate microbial community of the douzhi samples obtained from retail stores as well as the from subsequent fermentation experiments, 16S rRNA gene amplicon sequencing technology was employed by this study. In metagenomic research, although shotgun whole-genome sequencing can generate more detailed data and many other advantages, ribosomal RNA gene amplicon sequencing is chosen here due to its cost-efficiency, less sample input, lower computational requirement, and ability to avoid undesirable nucleic acid contamination. The remarkable versatility and constant advancements making it a cornerstone in various microbial research applications (Ranjan et al., 2016; Han et al., 2022). At each experimental stage, including sample handling, preservation, nucleic acid extraction, amplicon construction, sequencing, data processing, and interpretation, our research approach effectively achieved the goal to investigated microbial composition and dynamics in both douzhi and associated fermentation (Supplementary Figures S1–S3). The results demonstrated that the microbiota in all four stores is predominantly dominated by LAB, with five species, namely *Lactococcus lactis*, *Lactobacillus* sp., *Paucilactobacillus nenjiangensis*, *Latilactobacillus sakei* and *Leuconostoc lactis*, collectively comprising approximately 90% of the relative abundance in these stores. While some common genera found in fermented foods, such as *Bifidobacterium*, *Acetobacter*, and *Streptococcus*, were detected, but their overall presence was minimal (Figure 2). A prior investigation, utilizing plate isolation techniques, found that when douzhi is prepared following traditional methods, which include the incorporation of the microbiota from the previous fermentation stage which closely resembling homemade practices, the dominant microorganisms identified were *Lactococcus lactis* and *Leuconostoc citreum* (Ding et al., 2009). In contrast, the prominence of the other Lactobacillaceae species in our study was notably less significant. A more recent high-throughput 16S rRNA gene sequencing study demonstrated that the predominant bacterial genera in their sampled Beijing douzhi samples included *Lactococcus*, some Lactobacillaceae, *Streptococcus*, and *Klebsiella* (Xu et al., 2018). In this study, phylogenetic analysis indicates that there were minimal overall differences, both in alpha and beta diversity, among the four stores, especially compared with samples in fermentation experiments (Figures 2F,G). It can be inferred from these studies that the predominance of LAB in douzhi is a result of their adaptability to the fermentation matrices, environments, and processes. While various species of LAB may potentially serve as dominant strains within the douzhi microbiome, it is challenging for other microbes to undermine the predominant status of LAB during the fermentation process. Since traditional douzhi preparation involves backslopping, where a portion of the previous batch is used to inoculate the new batch. This practice, as recreated in this study, reinforces the LAB community and re-establish its dominance in each new fermentation cycle. The stability and resilience of the LAB population ensure the consistent quality and characteristics of douzhi in well controlled production.

Regarding the fermentation experiment, we observed notably different pH reduction patterns among the designated groups. Both group P and group L experienced the fastest and relatively lower final pH values compared to the control. Considering that this experiment did not equalize the initial total microbial counts and the inoculated LAB possessed a certain initial population, these variations are likely associated with the increased initial live microbial populations. Meanwhile, group M, which used a non-original matrix (skimmed milk) as the fermentation substrate, exhibited a much less pronounced pH decrease compared to other mung-bean based matrices during fermentation. The microbial population differences among groups were not significant, as all groups showed a consistent population increasing trend during the 24-h fermentation period. As for the microbial communities in the fermentation experiment, although the introduced strains, *Pediococcus pentosaceus* CGMCC 1.2695 (in group P) and *Lactiplantibacillus plantarum* CGMCC 1.1856 (in group L), were not native members of the microbiota, a significant proportion of the resident dominant microorganisms as well as the two inoculated LAB strains managed to maintain their relative abundance (Figure 4). Some adapted to these microbiota changes by reclaiming their ecological niches, while others experienced decrease or dramatic shift in their population during the fermentation process. Most intriguing phenomenon was observed in group M, all native dominant LAB species were severely affected by the fermentation process in skimmed milk, except for *Lactococcus lactis*, which was the only LAB species to maintain a significant population after 24 h. This may attribute to the variation in lactose utilization abilities of different strains, as lactose is the predominant energy source when milk is employed as the fermentation matrix (de Vos and Vaughan, 1994). Unexpectedly, one species, classified as *Escherichia* sp., exhibited a substantial increase from a very low percentage of the population to approximately 50% between the 12 and 24-h marks. While its exact species could not be conclusively determined, a NCBI Basic Local Alignment Search Tool (BLAST) analysis of the expanded amplicon sequence (410 bp) revealed a close match with 100% coverage and identification (data not shown) to 16S rRNA sequences of strains such as *Escherichia coli* O157:H7 str. Sakai and *Escherichia coli* str. K-12 substr. MG1655, hinting concerns about potential risks associated with this fermentation process. This result suggests that changing the fermentation matrix, even to another common LAB substrate like milk, can significantly alter the microbiota. Such changes can impact nutrient availability and environmental conditions, affecting microbial growth and activity. Therefore, any modifications to fermentation substrates should be carefully considered and tested to maintain product safety, quality, and the desired microbial community structure. Fermented dairy products and plant-based agricultural products share several common features in their fermentation processes. Concurrently, certain studies have revealed that dominant microorganisms in fermented dairy products such as kefir also encompass species like *Lactococcus*, Lactobacillaceae, and *Leuconostoc* (Tamang et al., 2020). Many studies in the field of fermented foods often explores the inclusion of various fermentation substrates as avenues for developing new fermented food products (Khurana and Kanawjia, 2007; Shahabbaspour et al., 2013; Soliman and Shehata, 2019; Shabbir et al., 2023). However, our study demonstrates that these fermented foods should not overlook food safety concerns, especially concerning the dynamics of high-risk and pathogenic microorganisms within complex microbial communities and fermentation matrices. Regarding the inoculated LAB, *Pediococcus pentosaceus* CGMCC 1.2695 and *Lactiplantibacillus plantarum* CGMCC 1.1856, our experiments revealed that *Lactiplantibacillus plantarum* CGMCC 1.1856 could maintain a relatively stable population, while the relative population of *Pediococcus pentosaceus* CGMCC 1.2695 significantly decreased during the 24-h fermentation period. Considering the lesser known physiological and metabolic characteristics of *Pediococcus pentosaceus*, along with its relatively less pronounced competitive advantage compared to other dominant LAB strains, while relevant research demonstrating its probiotic potential, our findings substantiate the possibility of introducing specific beneficial microorganisms into similar fermented food products (Jiang et al., 2021; Han et al., 2021a,b). These beneficial microbe supplementations may lead to potential effects such as increasing the initial total microbial count, accelerated pH reduction, and enhancement of the functional properties of the fermented food (Beena Divya et al., 2012; Blana et al., 2014).

The results in both the alpha and beta diversity analyses indicated that the addition of two types of LAB, particularly *Lactiplantibacillus plantarum* CGMCC 1.1856, had minimal impact on the overall microbial diversity (Figure 5). In contrast, the skimmed milk fermentation system (group M) had a significant and detrimental effect on alpha diversity. This is reasonable as only two dominant bacterial strains were preserved in the group M. In contrast, the addition of *Lactiplantibacillus plantarum* CGMCC 1.1856 appeared to largely maintain the beta diversity, considering that Lactobacillaceae are dominant microorganisms in the control group. PICRUSt (Phylogenetic Investigation of Communities by Reconstruction of Unobserved States) and its successor, PICRUSt2, are valuable tools for inferring the functional potential of microbial communities based on their taxonomic profiles derived from ribosomal RNA gene amplicon sequencing data (Langille et al., 2013; Douglas et al., 2020). PICRUSt2 was used in this study to gain insights into the metabolic capabilities of microbial communities of each group (Figure 6). It is apparent that, in beta diversity analyses on MetaCyc pathways and EC counts, three mung-bean-based fermentation matrices exhibit similar predicted functions throughout the entire fermentation process. In contrast, the microbiome of the skimmed milk fermentation exhibits markedly distinct functional profiles and significantly altered gene functions, primarily due to the presence of a unique dominant microorganism, *Escherichia* sp. By applying specific discrimination limits on enriched EC functions, we were able to illustrate that the LAB inoculation had a remarkable effect on the metabolic functions within group P and group L. In these groups, almost no decrease in metabolic functions was observed, when compared with control group. In fact, many EC functions, including pentose and glucuronate interconversions, porphyrin metabolism, and nicotinate and nicotinamide metabolism, were significantly enhanced in the microbiome at the 24-h mark. This observation highlights the significant influence of non-native LAB on the food fermentation process, particularly in douzhi, demonstrating their potential to introduce beneficial functions both throughout the fermentation and within the host’s gastrointestinal tract.

The comprehensive documentation of the fermented mung bean matrix through our metabolomic analysis provides a detailed view of the alterations in metabolite profiles due to microbial fermentation, both with and without the inoculation of LAB. Our study underscores the impact of LAB inoculation on the metabolic characteristics of douzhi. Although not dramatic, the obvious metabolite changes resulted from LAB addition highlights the unique metabolic capabilities that LAB introduce into the fermentation environment, which are not otherwise manifested by the indigenous douzhi microbial community. Our observations align with existing literature suggesting that LAB can significantly influence the metabolic pathways within fermented food matrices, thereby enhancing the bioavailability and concentration of key nutritional metabolites. In particular, the elevated levels of low molecular weight carbohydrates such as trehalose, melezitose, stachyose, and isoglobotriaose in the LAB samples point to a potential enhancement in the nutritional profile of the fermented mung bean matrix. Many of these carbohydrates are not only crucial for metabolism but may also play a role in the modulation of gut microbiota, which is beneficial for overall human health (Xi et al., 2020; Chen and Gibney, 2023; Aziz et al., 2024). Moreover, the significant differences in other nutritionally relevant compounds like galactinol, D-sorbitol, and caffeic acid further suggest that the addition of LAB could be strategically used to enhance the flavor and functional properties of fermented foods (Huang et al., 2018). Furthermore, the metabolite pathway enrichment analysis and corresponding differential abundance analysis, alone with metagenomic results, reveal that *Lactiplantibacillus plantarum* inoculation has a more pronounced effect on the metabolic pathways than *Pediococcus pentosaceus*.

In summary, our study discovered key findings into the microbial fermentation dynamics of douzhi microbiome, with a focus on the influence of specific LAB strains and altered matrix. The results demonstrated that douzhi sampled from representative chain outlets is predominantly composed of LAB and unveiled diverse effects when introducing LAB strains into the microbial community, whereas *Lactiplantibacillus plantarum* CGMCC 1.1856 maintained a stable presence, while *Pediococcus pentosaceus* CGMCC 1.2695 exhibited reduced relative abundance. Notably, the utilization of skimmed milk as a fermentation matrix led to significant alteration in microbial composition, marked by the dramatic increase of *Escherichia* sp., this observation emphasizes the need to consider the dynamics of high-risk microorganisms in complex microbial communities and fermentation matrices. The metabolomic analysis found that the LAB addition not only diversified the metabolites but also enhances the overall nutritional and functional properties of the mung bean matrix. The metagenomic and metabolomic analysis together revealed that LAB inoculation enhances various microbial and metabolic characteristics during the fermentation process.

This work provides key insights for optimizing fermentation processes to ensure consistent product quality and safety. Researchers can gain a deeper understanding of how the addition of specific microbial strains and variations in fermentation substrates influence the overall fermentation process. While for the industry, these findings offer a foundation for developing new fermentation strategies that meet consumer demands for health-promoting and safe food options. Future studies should explore the functional roles of identified LAB strains and refine fermentation processes to achieve optimal microbial community structures and product characteristics in douzhi and similar fermented foods. These efforts remain essential in both academic and industrial fields.

## Data availability statement

The amplicon sequencing data have been deposited in the NCBI BioProject database and are publicly accessible with the accession number PRJNA1026338. The data in this study are available upon request from the corresponding authors.

## Ethics statement

The manuscript presents research on animals that do not require ethical approval for their study.

## Author contributions

DH: Conceptualization, Formal analysis, Investigation, Software, Validation, Writing – original draft, Writing – review & editing. XB: Formal analysis, Methodology, Resources, Writing – review & editing. YFW: Formal analysis, Methodology, Writing – review & editing. XHL: Software, Validation, Writing – review & editing. KW: Software, Validation, Writing – review & editing. JC: Software, Validation, Writing – review & editing. XLL: Formal analysis, Writing – review & editing. ZY: Funding acquisition, Project administration, Resources, Writing – review & editing, Supervision. YBW: Conceptualization, Funding acquisition, Project administration, Supervision, Writing – review & editing.

## Footnotes

^1^http://www.hmdb.ca
