# Supplementary material for: The impact of lactic acid bacteria inoculation on the fermentation and metabolomic dynamics of indigenous Beijing douzhi microbial communities
Source: Front Microbiol. 2024 Jul 30;15:1435834. doi: 10.3389/fmicb.2024.1435834 (PMC11319256; doi:10.3389/fmicb.2024.1435834)
Supplement: Supplementary file 1 [file Image_1.pdf]

Supplementary material

The impact of lactic acid bacteria inoculation on the fermentation and metabolomic dynamics of indigenous Beijing douzhi microbial communities

Dong Han [1, 2], Xinyu Bao [1], Yanfang Wang [1], Khulood Fahad Alabbosh [3], Fahad Al-Asmari [4], Manal Y Sameeh [5], Xiaohong Liao [6], Ke Wang [6], Jian Chen [7], Xiaolong Li [1], Zhennai Yang [1, *], Yanbo Wang [1, *]

[1] Beijing Engineering and Technology Research Center of Food Additives, School of Food and Health, Beijing Technology and Business University, Beijing, China

[2] Key Laboratory of Food Bioengineering, (China National Light Industry), College of Food Science and Nutritional Engineering, China Agricultural University, Beijing, China

[3] Department of Biology, College of Science, University of Hail, Hail, Saudi Arabia

[4] Department of Food and Nutrition Sciences, College of Agricultural and Food Sciences, King Faisal University, Al-Hofuf, Saudi Arabia

[5] Department of chemistry, Al-Leith University College, Umm Al Qura University, Makkah, Saudi Arabia

[6] China National Light Industry Council, Beijing, China

[7] Food Safety Key Laboratory of Zhejiang Province, School of Food Science and Biotechnology, Zhejiang Gongshang University, Hangzhou, China

*** Correspondence:**

Zhennai Yang, yangzhennai@th.btbu.edu.cn

Yanbo Wang, wyb1225@163.com

Keywords: fermented foods, lactic acid bacteria, microbial community, Beijing douzhi, metabolomics

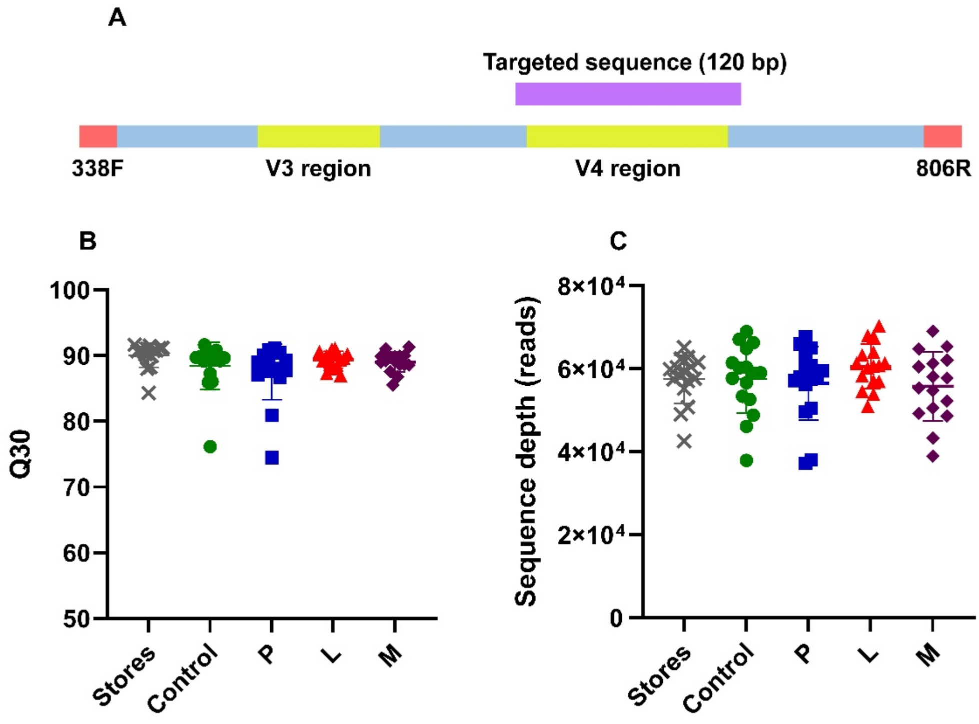

FIGURE S1. Quality assessment of amplicon sequencing data. **(A)** The PCR amplicon schematic illustration. The designated 120 bp target covering the V4 hypervariable region is highlighted in purple. Each region in the illustration is proportionate in length to represent a 469 bp amplicon. **(B)** The *Q*30 score, which represents the percentage of bases with a *Q* score greater than 30. **(C)** The sequencing depth of all samples, which were assessed by read counts within raw FASTQ file.

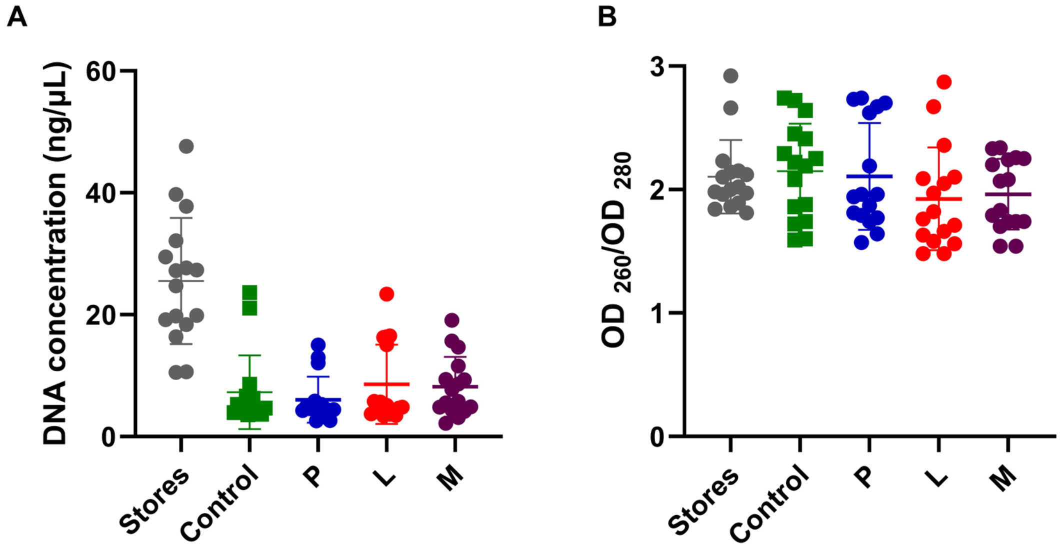

FIGURE S2. Extracted DNA quality assessment of all 80 samples in this study. **(A)** DNA concentrations. **(B)** OD260/OD280

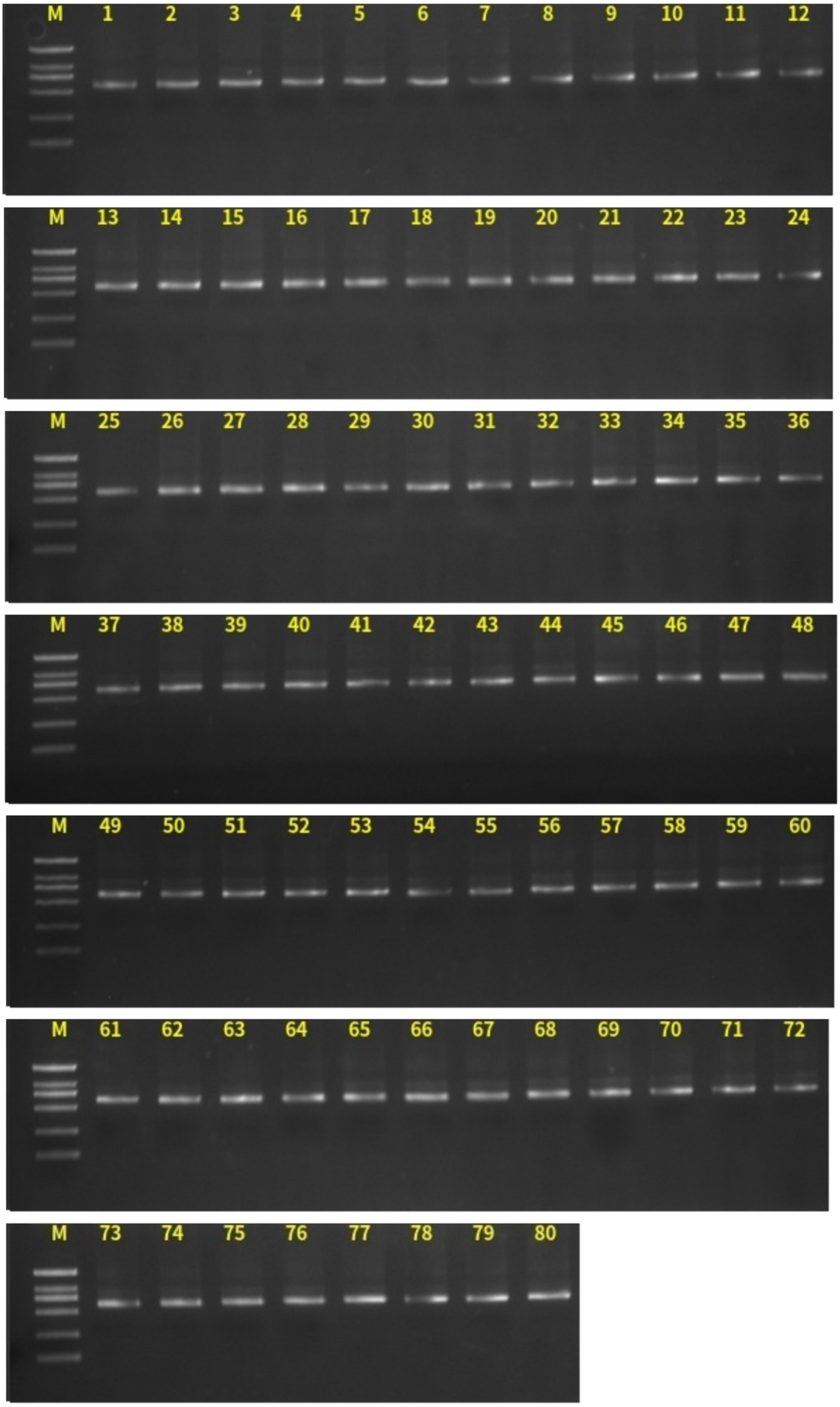

FIGURE S3. Analysis of 80 amplicon products using 1% agarose gel electrophoresis

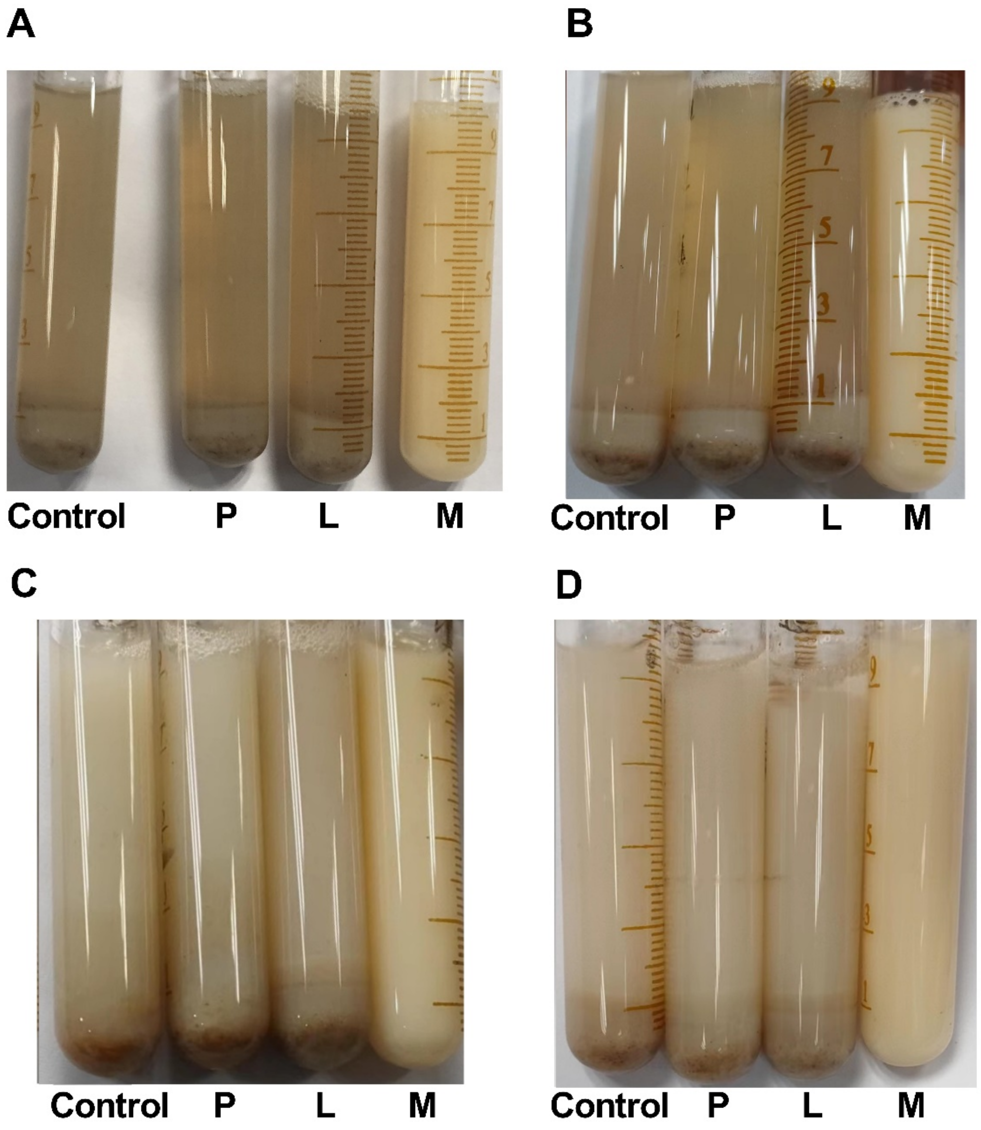

Figure

FIGURE S4 The visual changes after different fermentation durations in four groups: **(A)** 0 hour, **(B)** 2 hours, **(C)** 12 hours, and **(D)** 24 hours.

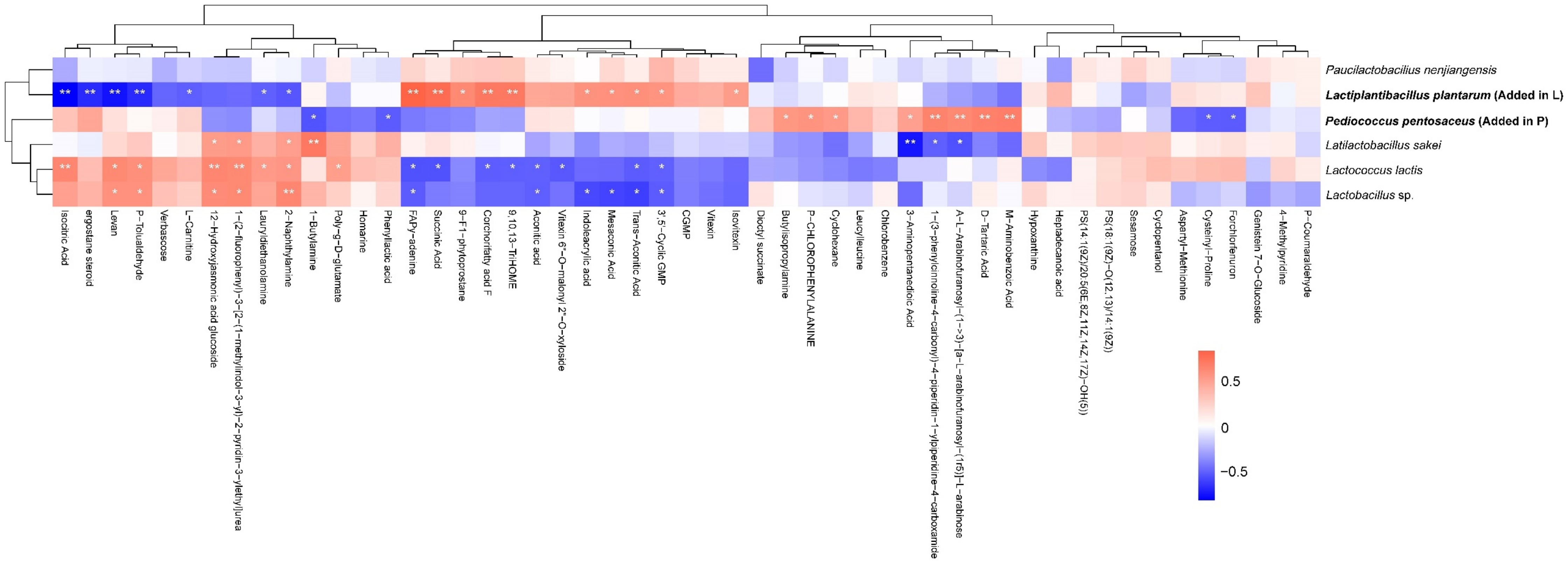

FIGURE S5 Cross-correlation analysis between the most abundant microbial species and metabolites after 24 hours of fermentation. The Pearson correlation coefficient (r) is calculated and presented, ranging from positive (red) to negative (blue) values. Differences are determined by FDR adjusted *p*-values, n = 4, ⋆: $p < 0.05$; ⋆⋆: $p < 0.01$.
